# A permeability-increasing drug synergizes with bacterial efflux pump inhibitors and restores susceptibility to antibiotics in multi-drug resistant *Pseudomonas aeruginosa* strains

**DOI:** 10.1038/s41598-019-39659-4

**Published:** 2019-03-05

**Authors:** Raquel Ferrer-Espada, Hawraa Shahrour, Betsey Pitts, Philip S. Stewart, Susana Sánchez-Gómez, Guillermo Martínez-de-Tejada

**Affiliations:** 1 University of Navarra, Department of Microbiology and Parasitology, Irunlarrea 1, 31008 Pamplona, Spain; 2 Navarra Institute for Health Research (IdiSNA), Pamplona, Spain; 3 Laboratory of Microbiology, Department of Life & Earth Sciences, Faculty of Sciences I, Lebanese University, Hadat campus, Beirut, Lebanon; 4 Platform of Research and Analysis in Environmental Sciences (PRASE), Doctoral School of Sciences and Technologies, Lebanese University, Hadat campus, Beirut, Lebanon; 5 Center for Biofilm Engineering, Montana State University, Bozeman, MT USA; 6 Bionanoplus S.L. Polígono Mocholí, Plaza Cein N°5, nave B14, 31110 Navarra, Spain; 7 Present Address: Wellman Center for Photomedicine, Massachusetts General Hospital, Harvard Medical School, 55 Fruit Street, Boston, MA 02114 USA

## Abstract

Resistance to antibiotics poses a major global threat according to the World Health Organization. Restoring the activity of existing drugs is an attractive alternative to address this challenge. One of the most efficient mechanisms of bacterial resistance involves the expression of efflux pump systems capable of expelling antibiotics from the cell. Although there are efflux pump inhibitors (EPIs) available, these molecules are toxic for humans. We hypothesized that permeability-increasing antimicrobial peptides (AMPs) could lower the amount of EPI necessary to sensitize bacteria to antibiotics that are efflux substrates. To test this hypothesis, we measured the ability of polymyxin B nonapeptide (PMBN), to synergize with antibiotics in the presence of EPIs. Assays were performed using planktonic and biofilm-forming cells of *Pseudomonas aeruginosa* strains overexpressing the MexAB-OprM efflux system. Synergy between PMBN and EPIs boosted azithromycin activity by a factor of 2,133 and sensitized *P. aeruginosa* to all tested antibiotics. This reduced several orders of magnitude the amount of inhibitor needed for antibiotic sensitization. The selected antibiotic-EPI-PMBN combination caused a 10 million-fold reduction in the viability of biofilm forming cells. We proved that AMPs can synergize with EPIs and that this phenomenon can be exploited to sensitize bacteria to antibiotics.

## Introduction

At the beginning of 2017, the World Health Organization issued for the first time in its history a global priority list of antibiotic-resistant bacteria^1^. This list included the 12 pathogens that pose the greatest threat to human health and its objective was to help in prioritizing the research and development of new antibiotic treatments. In particular, the document warned about the emergence of gram-negative pathogens that are resistant to multiple antibiotics, being carbapenem-resistant *P. aeruginosa* considered one of the critical priorities.

*P. aeruginosa* possesses both intrinsic and adaptive resistance to a wide variety of antimicrobials and frequently causes bacteremia, healthcare related pneumonia and urinary tract infections^2^. This organism is the most common bacterial species infecting the respiratory tract in cystic fibrosis patients^3^. The intrinsic mechanisms of resistance of *P. aeruginosa* include the low permeability of its outer membrane and the expression of numerous efflux systems that pump antibiotics out of the cell^4,5^. In addition, *P. aeruginosa* readily forms biofilms, surface-associated microbial communities that are extremely tolerant to antibiotics and immune system effectors. The ability of *P. aeruginosa* cells to form biofilms during infection facilitates its persistence inside the host^6^. Finally, *P. aeruginosa* rapidly develops resistance during anti-pseudomonal chemotherapy through overexpression of efflux pumps, loss of porins, alteration of drug targets or enzymatic modification of antibiotics.

Between 1940 and 1962, more than 20 different types of antibiotics were approved, whereas only 2 new classes of these drugs reached the market in the following 48 years^7^. As of May 2017, only 5 out of 33 antibiotics that are being developed for priority pathogens can be considered as novel agents^8^. Therefore, to control the continuous expansion of antimicrobial resistance, restoring the activity of existing antibiotics appears of critical importance.

Efflux pump activity contributes to reduced antibiotic susceptibility in both planktonic and biofilm cells^9–11^. *P. aeruginosa* has 12 resistance-nodulation-division (RND)-type efflux systems, being MexAB-OprM the best characterized^4^. This pump is constitutively expressed and exhibits an incredibly high ability to capture and extrude very structurally different antimicrobials including β-lactams, fluoroquinolones, macrolides, tetracyclines, trimethoprim, sulfamides and chloramphenicol^12^. The deletion of some regulatory genes such as *mexR, nalD* and *nalC* derepresses the system, thereby increasing bacterial resistance to its substrates^13^.

Two of the best characterized efflux pump inhibitors (EPIs) for Gram-negative bacteria are PAβN (Phenylalanine-Arginine β-Naphthylamide) and NMP (1-(1-naphthylmethyl)-piperazine)^14,15^. PAβN is a broad spectrum inhibitor of MexAB-OprM, MexCD-OprJ, and MexEF-OprN in *P. aeruginosa* and AcrABTolC in *E. coli*^15,16^. This EPI behaves itself as a substrate of efflux pumps binding in all likelihood to the transporter domain of these systems (the protein MexB in the case of MexAB-OprM)^15,17^. NMP is also a substrate of efflux pumps, although this EPI shows a pattern of antibiotic enhancing activity different from that of PAβN^15,18^.

Unfortunately, at high concentrations these and other EPIs are toxic to human cells and this fact hampered their clinical development up to now^15^.

We hypothesized that a possible way to decrease EPIs toxicity would be to co-administer them along with a membrane permeabilizing antimicrobial peptide (AMP). One of the most potent permeabilizers of this class for Gram-negative bacteria is polymyxin B nonapeptide (PMBN), a papain-cleaved derivative of polymyxin B (PMB)^19^. Interestingly, PMBN has an acute toxicity in mice of 43 mg/kg, almost five times lower than that of its parent compound, PMB, which is in clinical use^20^. Recently, PMBN was reported to be much less nephrotoxic than polymyxin E (colistin) and PMB in rats^21,22^.

PMBN has been shown to sensitize bacteria to antibiotics and here we investigated whether it also exhibits EPI-enhancing activity against planktonic and biofilm forming cells of *P. aeruginosa*^23–25^.

## Results

### Characterization of strains

We selected two *P. aeruginosa* strains that overexpress MexAB-OprM, namely LC1-6, a *nalB* mutant derivative of the wild type PAO1 strain^26^, and Ps4, a multidrug resistant clinical isolate previously characterized by our group^24^. As controls, we used two *P. aeruginosa* strains that do not overexpress MexAB-OprM, the wild type PAO1 and K1119^27^, a PAO1 derivate with a deletion in the efflux pump MexAB-OprM that abrogates its activity. To further characterize those strains, we determined the MIC of several antibiotics that had previously been described as substrates of the MexAB-OprM pump such as penicillins (piperacillin, amoxicillin, ampicillin and ticarcillin), third generation cephalosporines (ceftazidime), monobactams (aztreonam), macrolides (azithromycin and erithromycin), tetracyclines (doxycycline, tetracycline) and quinolones (ciprofloxacin, levofloxacin and ofloxacin)^12,28^. The susceptibility of the strains to PMBN and two EPIs (NMP and PAβN) was also assessed.

**Figure 1:**
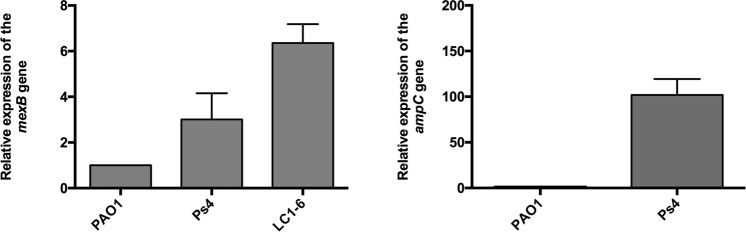
Strain LC1-6 overexpresses the gene *mexB* and Ps4 overexpresses the genes *mexB* and *ampC*, as determined by RT-qPCR. Results shown are the means ± standard deviation of three independent experiments where each strain was tested in triplicate wells (n = 9). The value of *P. aeruginosa* PAO1 was 1.

As shown in Table 1, Ps4 susceptibility profile was compatible with overexpression of MexAB-OprM and resembled that of LC1-6. These results confirmed previous observations made by our group in these organisms^24^. In agreement with their MICs, RT-qPCR analysis confirmed that Ps4 and LC1-6 overexpressed *mexB* (Fig. 1), although levels of this gene transcript in the mutant LC1-6 were markedly superior. Additional RT-qPCR based characterization revealed that Ps4 also overproduced the cephalosporinase AmpC (Fig. 1). This fact explains in all likelihood the increased resistance to some β-lactams (i.e. piperacillin, ticarcillin and ceftazidime) displayed by Ps4 in comparison with the other strain. The relative insensitivity of PS4 to levofloxacin was probably due to a mutation in codon 83 of *gyrA* (83/(ACC:ATC)/Thr: Ile), as previously reported^29^. On the other hand, all the strains were highly resistant both to PMBN and EPIs (Table 1).

**Table 1:** Antimicrobial susceptibity of *Pseudomonas aeruginosa* strains used in this work.

Antimicrobials			MIC (μg/mL)			
			Ps4	LC1-6	PAO1	K1119
β-lactams	Penicillins	Piperacillin	256 (R^a^)	16 (S^b^)	4 (S)	0.5 (S)
		Amoxicillin	>512	>512	>512	>512
		Ampicillin	>512	>512	>512	>512
		Ticarcillin	256 (R)	128 (R)	16 (S)	≤1 (S)
	Cephalosporins	Ceftazidime	64 (R)	4 (S)	2 (S)	4 (S)
	Monobactams	Aztreonam	16 (I^c^)	32 (R)	32 (R)	≤1 (S)
	Carbapenems	Imipenem	S	nd^d^	S	nd
		Meropenem	S	R	S	nd
Macrolides	Azithromycin		128	128	128	32
	Erithromycin		256	256	128	32
Tetracyclines	Doxycycline		64	64	4	2
	Tetracycline		64	32	8	≤1
Quinolones	Levofloxacin		16(R)	2 (S)	0.25 (S)	0.12 (S)
	Ofloxacin		16 (R)	4 (I)	1 (S)	0.5 (S)
	Ciprofloxacin		4 (R)	≤1 (S)	≤1 (S)	≤1 (S)
Antimicrobial Peptides	PMBN^e^		>512	>512	>512	>512
Efflux pump inhibitors	NMP^f^		128	64	64	64
	PaβN^g^		>512	>512	>512	>512

^a^Resistant, ^b^Susceptible and ^c^Intermediate according to Clinical and Laboratory Standards Institute (CLSI) guidelines. ^d^Not determined. ^e^Polymyxin B Nonapeptide. ^f^1-(1-naphthylmethyl)-piperazine. ^g^L-Phe-L-Arg-β-naphthylamide dihydrochloride.

Finally, the ability of the selected strains to attach to surfaces and form biofilms was measured by using a microplate assay. These experiments demonstrated that LC1-6 had a very low adherent capacity, as opposed to Ps4 and K1119, which consistently formed very dense biofilms.

### Screening of PMBN-dependent EPI potentiation on *P. aeruginosa* LC1-6 by the checkerboard method

For these planktonic cell assays, we used the strain with the highest level of MexAB-OprM overexpression, LC1-6. As shown in Table 2, LC1-6 showed a moderate to high level of resistance to the majority of antibiotics tested (see column “MIC alone”). Interestingly, concentrations as low as 1 μg/mL of both PAβN and PMBN reduced the MIC value of Azithromycin more than 2,000 times (i.e. from 128 μg/mL to 0.06 μg/mL). In contrast, PAβN by itself (i.e. without PMBN) failed to sensitize LC1-6 to Azythromycin even at the highest concentration of EPI tested (see the upper and leftmost panel of Supplementary Table 1a). In agreement with these results, the parameter used to quantify antibiotic-enhancing activity, FICI, indicated that indeed the combination Azithromycin/PAβN/PMBN was extremely synergistic (FICI = 0.002; Table 2

**Table 2:** Summary of synergy testing conducted on *Pseudomonas aeruginosa* LC1-6 by the checkerboard method.

EPI^a^	AB^b^	Antibiotic Mic (µg/mL)^c^		Enhancement	FICI^e^			
		alone	+EPI + PMBN^g^	factor^d^	AB/EPI/PMBN	AB/EPI	EPI/PMBN	AB/PMBN
**PAβN** ^**f**^								
	Azithromycin	128	0.06 (1/1)	2133	**0.002**	2.00	1.00	**0.01**
	Doxycycline	64	0.12 (1/1)	533	**0.004**	1.00	1.00	**0.25**
	Levofloxacin	2	0.007 (2/1)	286	**0.006**	1.00	1.00	**0.50**
	Ceftazidime	4	0.015 (2/1)	267	**0.007**	1.00	1.00	1.00
	Piperacillin	16	0.06 (2/1)	267	**0.007**	1.00	1.00	1.00
	Aztreonam	32	2 (4/1)	16	**0.07**	0.51	1.00	1.00
**NMP** ^**h**^								
	Azithromycin	128	0.50 (1/1)	256	**0.02**	1.02	1.00	**0.01**
	Doxycycline	64	1 (1/1)	64	**0.03**	1.02	1.00	**0.25**
	Levofloxacin	2	0.12 (4/1)	17	**0.12**	1.06	1.02	**0.50**
	Ceftazidime	4	1 (8/1)	4	**0.38**	1.06	1.03	1.00
	Piperacillin	16	4 (16/1)	4	**0.50**	1.25	1.06	1.00
	Aztreonam	32	8 (16/1)	4	**0.50**	0.75	1.06	1.00

^a^Efflux pump inhibitor; ^b^Antibiotic; ^c^Minimum inhibitory concentration (µg/mL) of antibiotic alone or in combination with EPI and polymyxin B nonapeptide (the concentration of EPI and PMBN, respectively, present in the triple combination is shown in parenthesis); ^d^Maximum number of times that the antibiotic MIC is reduced in the presence of EPI and PMBN; ^e^Fractional inhibitory concentration index of the combination rendering maximum enhancement and all its possible double variants (combinations were considered as synergistic if FICI ≤ 0.5, indifferent if 0.5< FICI ≤ 4, or antagonistic if FICI > 4) (see Material and Methods); ^f^*L-Phe-L-Arg-β-naphthylamide dihydrochloride*; ^g^Polymyxin B Nonapeptide; ^h^*1-(1-naphthylmethyl)-piperazine*. Synergistic combinations are indicated in bold.

), a value much lower than that considered as the cutoff for synergism (FICI ≤ 0.5). These results demonstrate that a concentration as low as 1 μg/mL of PAβN causes a dramatic sensitization of strain LC1-6 to Azythromycin in the presence of minute amounts of PMBN.

Although the enhancement factor obtained with other antibiotics was less pronounced, the synergism among the three components was even more clear than that shown for Azythromycin. For instance, a combination of PAβN and PMBN similar as that used previously (2 and 1 μg/mL of each compound) sufficed to lower more than 267 times the MIC of Ceftazidime and to reach a FICI value of 0.007 (Table 2 and Supplementary Table 1a). Importantly, in this case none of the three double combinations that could be composed with those three components (Ceftazidime/PAβN, Ceftazidime/PMBN and PMBN/PAβN) was close to be synergistic (FICI = 1). In contrast, Azithromycin acted in synergy with PMBN, although with much less potency than in the presence of the third component PAβN (0.01 for the double vs. 0.002 for the triple combination; Table 2). Results obtained with Levofloxacin and Piperacillin were very similar to those of Ceftazidime, whereas enhancement was less potent (16-fold reduction in MIC; FICI = 0.07) in the case of Aztreonam.

To study whether the observed potentiation could be achieved using EPIs other than PAβN, we repeated the assays using the structurally unrelated inhibitor NMP. As shown in the lower panel of Table 2, antibiotic activity was also enhanced in the presence of NMP but much less than in the case of PAβN, as reflected by significantly lower enhancement factors and higher FICI values (see also Supplementary Table 1b).

### Evaluation of selected triple combinations on *P. aeruginosa* Ps4 by the checkerboard method

The most potent triple combinations were selected and tested against the multiresistant clinical strain Ps4 using the checkerboard method. As mentioned before, this strain not only overexpresses MexAB-OprM but also overproduces the cephalosporinase AmpC (Fig. 1).

In the absence of PMBN, the inhibitor PAβN had to be added at the highest concentration tested (16 μg/mL) to cause a rather modest reduction in the MIC of two antibiotics (Azithromycin and Doxycycline) (Supplementary Table 2). On the contrary, the addition of 1 μg/mL of PMBN together with PAβN enhanced 512 times and 128 times the antimicrobial activity of azithromycin (from 128 to 0.25 μg/mL) and doxycycline (from 64 to 0.5 μg/mL), respectively (Table 3). Notably, the enhancement obtained with the β-lactam antibiotics ceftazidime and piperacillin was of much lower level (16 times, at most), a likely consequence of AmpC overexpression in the test strain (Fig. 1). Despite this fact, FICIs obtained with those drugs were indicative of a good synergistic activity (0.07 and 0.13, respectively) suggesting that EPI may be effective in strains with a high level of expression of β-lactamases thereby contradicting reported claims^14^

**Table 3:** Summary of synergy testing conducted on *Pseudomonas aeruginosa* Ps4 by the checkerboard method.

EPI^a^	AB^b^	ANTIBIOTIC MIC (µg/mL)^c^		Enhancement	FICI^e^			
		alone	+EPI + PMBN^g^	factor^d^	AB/EPI/PMBN	AB/EPI	EPI/PMBN	AB/PMBN
**PAβN** ^**f**^								
	Azithromycin	128	0.25 (4/1)	512	0.01	1.00	1.00	0.50
	Doxycycline	64	0.50 (4/1)	128	0.01	1.00	1.00	0.50
	Ceftazidime	64	4 (8/1)	16	0.07	1.00	1.00	0.50
	Piperacillin	256	32 (4/1)	8	0.13	1.00	1.00	0.50
**NMP** ^h^								
	Azithromycin	256	4 (2/1)	64	0.03	1.01	1.06	0.25
	Doxycycline	64	0.25 (1/1)	256	0.01	1.00	1.06	0.50
	Ceftazidime	64	0.25 (4/1)	256	0.04	0.53	1.06	0.50
	Piperacillin	256	8 (1/1)	32	0.04	0.51	1.06	0.50

^a^Efflux pump inhibitor; ^b^Antibiotic; ^c^Minimum inhibitory concentration (µg/mL) of antibiotic alone or in combination with EPI and polymyxin B nonapeptide (the concentration of EPI and PMBN, respectively, present in the triple combination is shown in parenthesis); ^d^Maximum number of times that the antibiotic MIC is reduced in the presence of EPI and PMBN; ^e^Fractional inhibitory concentration index of the combination rendering maximum enhancement and all its possible double variants (combinations were considered as synergistic if FICI ≤ 0.5, indifferent if 0.5 < FICI ≤ 4, or antagonistic if FICI > 4) (see Material and Methods); ^f^*L-Phe-L-Arg-β-naphthylamide dihydrochloride*; ^g^Polymyxin B Nonapeptide; ^h^*1-(1-naphthylmethyl)-piperazine*. Synergistic combinations are indicated in bold.

.

When the antibiotics were combined with NMP and PMBN, synergistic combinations were also observed (Table 3, Supplementary Table 2).

Although all the triple combinations tested were found to be synergistic (Table 3), the FICIs obtained in Ps4 with PAβN were approximately an order of magnitude higher (i.e less potent) than the equivalent ones measured in LC1-6. In contrast, the FICIs obtained in Ps4 with NMP were more potent than the ones determined in LC1-6. As previously shown with LC1-6, PAβN and NPM synergized in strain Ps4 neither with antibiotics nor with PMBN (see columns AB/PAβN and PAβN/PMBN in Table 3). However, in this strain, PMBN slightly synergized with all the antibiotics in the absence of the EPI, although the triple combinations improved notably (from 4 to 50 times) the FICI values of the double ones in all the cases.

### Evaluation of selected triple combinations on *P. aeruginosa* K1119 by the checkerboard method

The most potent triple combinations were also tested in K1119, a *mexAB-oprM* deletion mutant. As somewhat expected in a mutant lacking one of the major efflux pumps, exposure to PAβN even at the highest concentrations had no effect on K1119 antibiotic susceptibility (Supplementary Table 3a). In contrast, the other EPI, NMP, showed a significant antibiotic enhancing activity by itself when added at medium to high concentrations (Supplementary Table 3b).

Apart from the Doxyxycline/PMBN and Piperacillin/NMP combinations, none of the other double combinations were synergistic (Table 4). The FICI values obtained with the triple combinations paralleled the lowest FICIs obtained with the double combinations and were not improved by addition of a triple component except for the azithromycin/PMBN/NMP triple combination (Table 4, Supplementary Table 3

**Table 4:** Summary of synergy testing conducted on *Pseudomonas aeruginosa* K1119 by the checkerboard method.

EPI^a^	AB^b^	ANTIBIOTIC MIC^c^ (µg/mL)		Enhancement factor^d^	FICI^e^			
		alone	+EPI + PMBN^g^		AB/EPI/PMBN	AB/EPI	EPI/PMBN	AB/PMBN
**PAβN** ^**f**^								
	Azithromycin	32	16(1/1)	2	0.502	1.001	1	0.501
	Doxycycline	2	0.125 (1/1)	16	0.064	1.016	1	0.063
	Piperacillin	0.5	0.5 (1/1)	1	1.002	1.016	1	1.001
**NMP** ^h^								
	Azithromycin	32	0.03(4/1)	1066	0.064	0.563	1.06	0.501
	Doxycycline	2	0.06 (2/1)	32	0.063	1.031	1.06	0.063
	Piperacillin	0.5	0.03 (8/1)	16	0.188	0.188	1.06	1.001

^a^Efflux pump inhibitor; ^b^Antibiotic; ^c^Minimum inhibitory concentration (µg/mL) of antibiotic alone or in combination with EPI and polymyxin B nonapeptide (the concentration of EPI and PMBN, respectively, present in the triple combination is shown in parenthesis); ^d^Maximum number of times that the antibiotic MIC is reduced in the presence of EPI and PMBN; ^e^Fractional inhibitory concentration index of the combination rendering maximum enhancement and all its possible double variants (combinations were considered as synergistic if FICI ≤ 0.5, indifferent if 0.5 < FICI ≤ 4, or antagonistic if FICI > 4) (see Material and Methods); ^f^*L-Phe-L-Arg-β-naphthylamide dihydrochloride*; ^g^Polymyxin B Nonapeptide; ^h^*1-(1-naphthylmethyl)-piperazine*. Synergistic combinations are indicated in bold.

).

### Evaluation of selected triple combinations on *P. aeruginosa* PAO1 by the checkerboard method

The most potent triple combinations were also tested in PAO1, a strain expressing wild type levels of MexAB-OprM. In contrast to K1119, the PAβN inhibiting effect was clearly detectable in PAO1 with the three tested antibiotics (Supplementary Table 4a). NMP was also able to enhance antibiotic activity in the absence of AMP. Azythromycin and Doxycycline synergized with EPIs and PMBN. However, this enhancement can be attributed in its totality to the synergistic activity detected between the antibiotics and the AMP. On the other hand, the synergism observed between piperacillin, PMBN and both EPIs was higher than any of the other possible double combinations (Table 5, Supplementary Table 4

**Table 5:** Summary of synergy testing conducted on *Pseudomonas aeruginosa* PAO1 by the checkerboard method.

EPI^a^	AB^b^	ANTIBIOTIC MIC^c^ (µg/mL)		Enhancement factor^d^	FICI^e^			
		alone	+EPI + PMBN^g^		AB/EPI/PMBN	AB/EPI	EPI/PMBN	AB/PMBN
**PAβN** ^**f**^								
	Azithromycin	256	4 (1/1)	64	0.018	1.001	1	0.017
	Doxycycline	4	0.03 (1/1)	133	0.01	1.001	1	0.009
	Piperacillin	4	0.5 (1/1)	8	0.127	0.516	1	0.251
**NMP** ^h^								
	Azithromycin	256	4 (1/1)	64	0.032	1.016	1.06	0.017
	Doxycycline	4	0.03 (1/1)	133	0.024	1.016	1.06	0.009
	Piperacillin	4	0.06 (2/1)	63	0.048	1.031	1.06	0.251

^a^Efflux pump inhibitor; ^b^Antibiotic; ^c^Minimum inhibitory concentration (µg/mL) of antibiotic alone or in combination with EPI and polymyxin B nonapeptide (the concentration of EPI and PMBN, respectively, present in the triple combination is shown in parenthesis); ^d^Maximum number of times that the antibiotic MIC is reduced in the presence of EPI and PMBN; ^e^Fractional inhibitory concentration index of the combination rendering maximum enhancement and all its possible double variants (combinations were considered as synergistic if FICI ≤ 0.5, indifferent if 0.5 < FICI ≤ 4, or antagonistic if FICI > 4) (see Material and Methods); ^f^*L-Phe-L-Arg-β-naphthylamide dihydrochloride*; ^g^Polymyxin B Nonapeptide; ^h^*1-(1-naphthylmethyl)-piperazine*. Synergistic combinations are indicated in bold.

).

### Kinetics of inhibition of *P. aeruginosa* Ps4 by triple combinations

**Figure 2:**
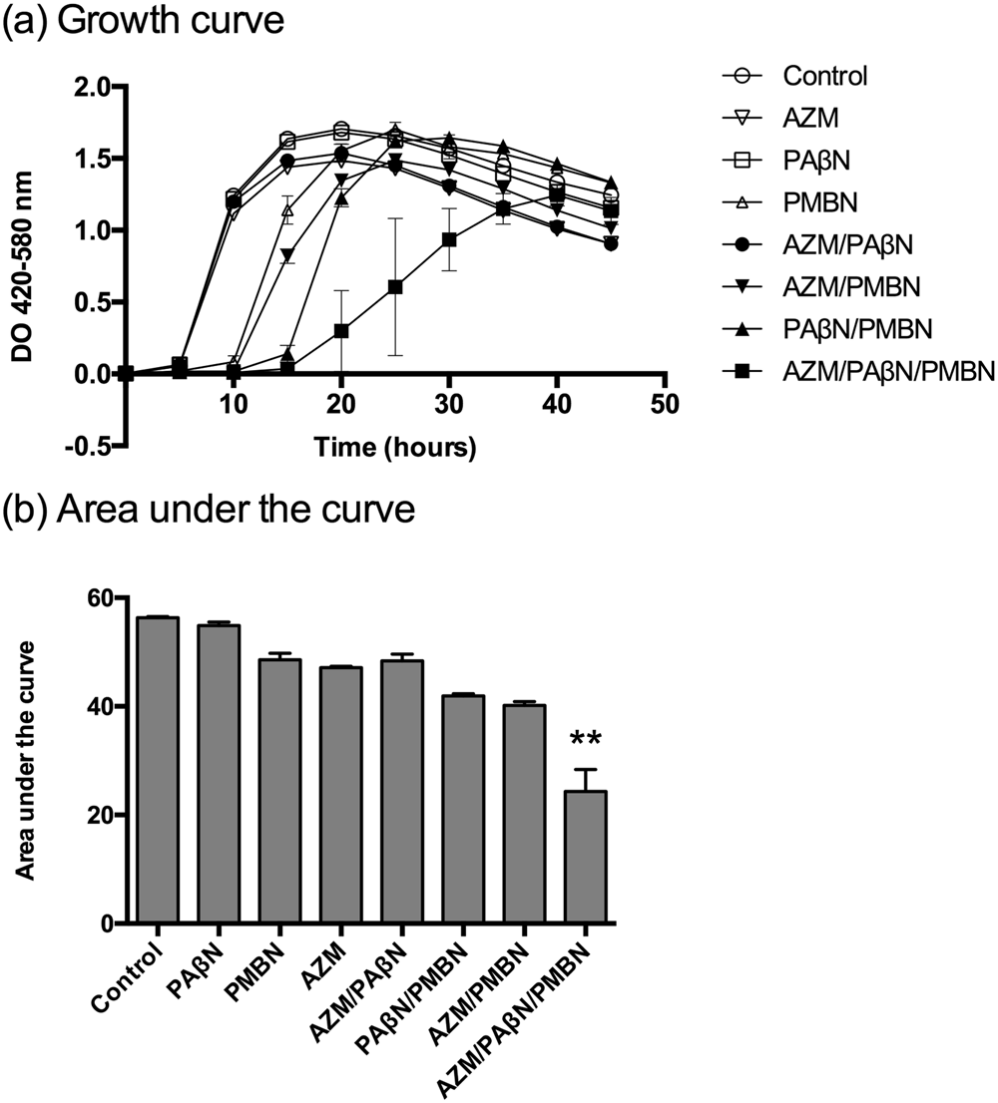
PMBN sensitizes a MexAB-OprM overexpressing *Pseudomonas aeruginosa* clinical strain (Ps4) to PAβN and an antibiotic substrate of MexAB-OprM (azithromycin), as determined by turbidimetry (Bioscreen C). (**a**) At time 0, cultures were exposed to the indicated antimicrobial combinations and incubated with shaking at 37 °C in an optical analyzer that automatically monitors optical density at regular intervals. The inoculum and the culture medium (MHCA) were the same as those used to determine the MIC. AZM: azithromycin (0.25 μg/mL); PAβN: L-Phe-L-Arg-β-naphthylamide (4 μg/mL); PMBN (1 μg/mL). (**b**) Area under the curve during the first 45 h of growth of indicated cultures (panel (a)). Results shown are the means ± standard deviation of three independent experiments where each concentration was tested in triplicate wells (n = 9). Data were analyzed using Kruskal Wallis test with multiple comparisons and statistical differences between the culture treated with the triple combination and the untreated control were very significant (**p = 0.0077). Alpha = 0.05.

To further discriminate the contribution of each component of the combination to the global antimicrobial activity observed, growth inhibition assays were performed using the automated optical analyzer Bioscreen C. For these assays, we selected the combination that displayed the highest potency against the two MexAB-OprM overexpressing strains, namely azithromycin/PAβN/PMBN. As shown in Fig. 2a, this combination was the only one capable of completely inhibiting the growth of Ps4 during the first 15 h. The removal of any compound from that combination resulted in a significant loss of the inhibitory capacity. To quantify the growth inhibition due to each treatment we determined the corresponding area under the curve (AUC). In agreement with previous results, comparison of the AUCs of the different treatments revealed that only the triple combination azithromycin/PAβN/PMBN could inhibit in a very significant way (**p < 0.01) the growth of Ps4 (Fig. 2b). Taken together, these results demonstrate that a permeabilizing agent at subinhibitory concentrations can synergize with EPIs and sensitize efflux pump overexpressing strains of *P. aeruginosa* to antibiotics substrates of those EPIs.

### Enhancement of EPIs by PMBN in biofilms of *P. aeruginosa* Ps4 grown in the CDC biofilm reactor

**Figure 3:**
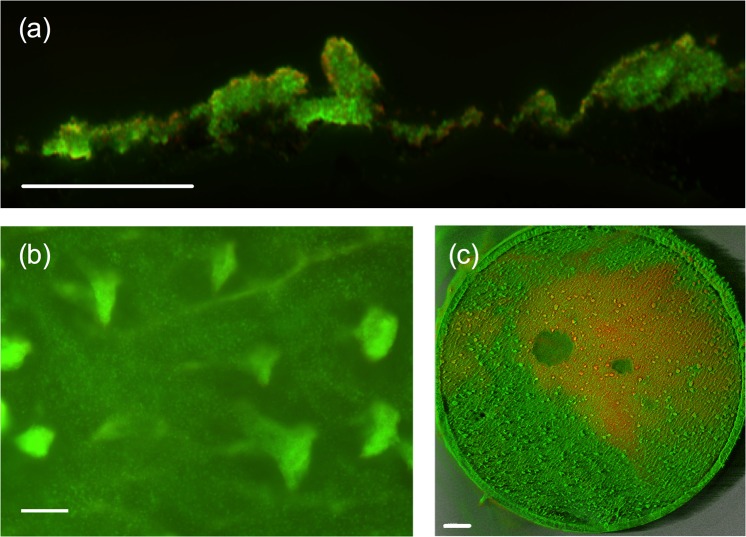
When grown under turbulent conditions in the CDC reactor, *Pseudomonas aeruginosa* Ps4 forms dense biofilms that stain green with the LIVE/DEAD BacLight kit. (**a**) Cryosectioning fluorescence microscope image taken at 20x magnification. (**b**) Top view at low magnification (10x; fluorescence microscopy) of the surface of a coupon coated with a biofilm layer. (**c**) Confocal laser scanning microscope image of the coupon surface at 1.5x magnification, Scale bars length is 100 μm in (**a**) and (**b**) and 1000 μm in (**c**).

Previously, we showed that Ps4 could form biofilms under static growth conditions in microplates. However, to try to mimic *in vivo* conditions we also confirmed that this strain could also form biofilms when exposed to a turbulent flow in the CDC reactor. In this device, the biofilm develops on the surface of small disks called coupons that are constantly bathed in fresh culture medium. Biofilm formation was assessed by fluorescence and confocal microscopy on coupons stained with the LIVE/DEAD BacLight kit. Analysis of the surface or cryosections of coupons grown under these conditions revealed the characteristic morphology of biofilms with abundant finger-like projections protruding from the surface. (Fig. 3)

**Figure 4:**
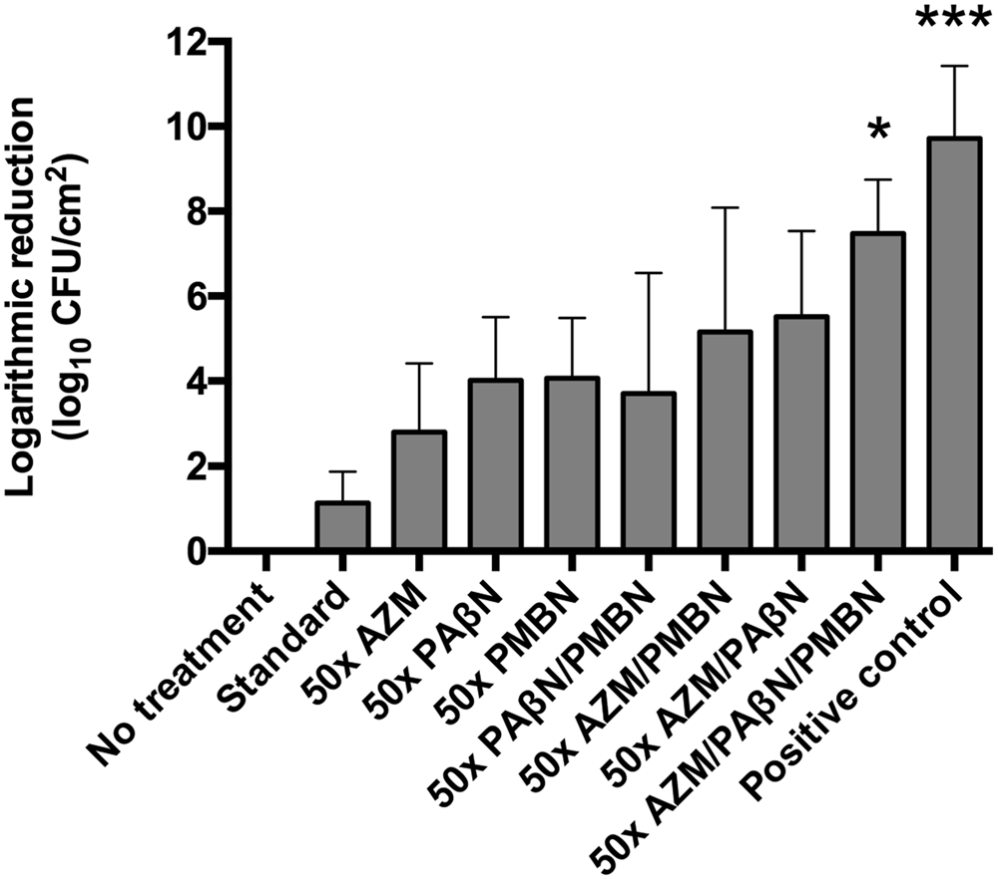
The three components of the triple combination (antibiotic/EPI/PMBN) are necessary to reduce the number of biofilm cells of *Pseudomonas aeruginosa* Ps4 grown in the CDC biofilm reactor. After 24 h of incubation under continuous flow, coupons coated with 48 h mature biofilms were removed from the reactor, washed and exposed at 37 °C for 72 h (with renewal of the solution every 24 h) to either a positive control of bactericidal activity (chlorine 1,000 μg/mL), a standard treatment for Gram-negative biofilm eradication (ceftazidime 5,000 μg/mL) or the indicated antimicrobial combinations at 50 times their planktonic MIC in phosphate buffer (planktonic MIC = AZM (0.25 μg/mL); PAβN (4 μg/mL); PMBN (1 μg/mL)). Then, biofilms were detached from coupons by using mechanical means, homogenized by sonication and viable bacteria were enumerated by colony counting. Finally, the logarithmic reduction of viable cells caused by each treatment was calculated using untreated coupons as reference controls of viability. AZM: Azithromycin; PAβN: L-Phe-L-Arg-β-naphthylamide; PMBN: Polymyxin B nonapeptide. Results shown are the means ± standard deviation of two independent experiments where each condition was tested in duplicate coupons (n = 4). Differences between treatments were analized with a One-Way-ANOVA followed by Tukey’s multiple comparison test. Differences were highly significant for the positive control (***p = 0.0002) or significant for the triple combination (*p = 0.0156) compared to the untreated control. Alpha = 0.05.

To study if PMBN-mediated enhancement of EPI was also efficient against *P. aeruginosa* biofilms we treated coupons with the selected triple combination at 50 times its planktonic MIC (i.e. Azithromycin (12.5 μg/mL), PAβN (200 μg/mL) and PMBN (50 μg/mL)). In theory, Azithromycin is particularly well-suited for anti-biofilm therapy because it has been reported to interfere with the quorum sensing signals mediated by *lasI*.^6,30–33^. As shown in Fig. 4, the selected combination reduced in a significant way (*p < 0.05) bacterial viability (7.5 logarithms) of a 48h-old biofilm, although the positive control of bactericidal activity (chlorine at 1,000 μg/mL) showed more potent activity. Similar to our observations with planktonic cells of Ps4, removal of any component of the combination significantly decreased its anti-biofilm activity.

Our results indicate that, at 50 times its planktonic MIC, the experimental combination reduces more than 10,000,000 times the viability of mature biofilms formed by Ps4 and that EPI enhancement by PMBN seems to play a role in this bactericidal activity.

## Discussion

In the present study, we have demonstrated for the first time that a permeabilizing agent at subinhinbitory concentrations is able to sensitize strains that overexpress MexAB-OprM to a combination of a substrate antibiotic and an EPI, being the combination unsuccessful in the absence of the permeabilizing agent.

Specifically, we found that at subinhibitory concentrations, PMBN-mediated potentiation of PAβN resulted in sensitization to antibiotics by factors of up to 2,133 in the mutant strain *P. aeruginosa* LC1-6 (Table 2; Supplementary Table 1a). The activity of the inhibitor NMP in combination with antibiotics was also enhanced by PMBN (Table 2; Supplementary Table 1b), but to a lesser extent than when PAβN was used in that same strain. This observation is in agreement with previous reports showing that PAβN has more efflux pump inhibiting potency than NMP^15,34,35^. Accordingly, the maximum enhancement achieved by NMP was of 256 times when this EPI was combined with Azithromycin (from 128 μg/mL to 0.5 μg/mL; FICI_min_ = 0.02). It is important to note that the level of enhancement achieved by PMBN with the antibiotics Azithromycin and Doxycycline was higher than with the other antibiotics (i.e. in the absence of the EPI; first rows of Supplementary Table 1a,b), and this correlated with the potency of the sensitization obtained with each triple combination. This fact reflects in all likelihood the mechanism used by each antibiotic to cross the outer membrane of *P. aeruginosa*, namely the lipid-mediated pathway for azithromycin and doxycycline and the porin-mediated for the rest^36^. As a consequence, antimicrobials using the former pathway heavily rely on permeabilization by PMBN to gain access to their intracelullar target, whereas those of the second group had a much lower dependence of this mechanism (Tables 2–5). Nevertheless, our results demonstrate that all triple combinations when tested in the MexAB-OprM overexpressing strains LC1-6 and Ps4, strictly depend on EPIs to achieve their maximum sensitization level. On the other hand, when the test strains do not overexpress MexAB-OprM, such as in PAO1 or K1119, the observed synergistic activity can be attributed to either the antibiotic/PMBN or antibiotic/EPI combination.

The higher expression of *mexAB-oprM* in LC1-6 compared to Ps4 might explain why NMP is more potent in the latter strain.

The observed Antibiotic/NMP synergistic activity in the strain K1119 (Table 4; Supplementary Table 3b) seems to indicate a hypothetic NMP activity in other efflux pumps different from MexAB-OprM whose expression might be induced by antibiotic exposure (Supplementary Table 3). This activity would go unnoticed in the strain LC1-6 because NMP concentration would be insufficient to inhibit the elevated MexAB-OprM levels expressed by this strain (Supplementary Table 1).

In addition to its inhibitory action, it has been reported that PAβN acts as a weak permeabilizer when used at sufficiently high concentrations (20 μg/mL), whereas its EPI activity is observable at much lower concentrations (1,25 μg/mL)^37,38^. In contrast, the other EPI used in our assays, NMP, has no reported permeabilizing activity. Since PAβN synergized with antibiotics in our assays at concentrations as low as 1 μg/mL, this activity results in all likelihood from the inhibition of efflux pumps. In agreement with this, enhancement of antibiotic activity by the other EPI, NMP, was also detected at low concentrations (1–4 μg/mL).

In the presence of the inhibitor PAβN, the triple combinations exhibited a loss of activity at concentrations higher than or equal to 4–8 μg/mL of the EPI (Supplementary Table 1a). This might be due to aggregation of PAβN, which is strongly cationic at neutral pH and could be displaced by other cations (Mg^2+^ and Ca^2+^ present in culture medium) or by the polycationic agent PMBN^14^. In contrast, this saturation effect was not observed when using the inhibitor NMP (Supplementary Table 1b), which has much less cationic character^39^. Nevertheless, no solubility problems (e.g. the presence of insoluble precipitates) were detected during the course of this study. Alternatively, this loss of activity could be due to an Eagle effect of PAβN. The phenomenon has been described as a paradoxical effect of drugs where more drug kills less and apparently is not related to solubility^40,41^.

In addition, we showed that the potentiation of EPIs by PMBN caused a 10 million-fold reduction in biofilm viability in a multidrug clinical isolate of *Pseudomonas*. In agreement with this conclusion, other authors reported that efflux pumps are highly active in young (<4 day-old) bacterial biofilms^10,42^, like ours and MexCD-OprJ, MexAB-OprM and a novel ABC transporter of *P. aeruginosa* were shown to be involved in biofilm resistance to Azithromycin^9–11^. Besides, there is evidence supporting that biofilms are susceptible to antibiotic sensitization by permeabilizers of different nature including farnesol^43^, EDTA^44^, cholic acid^45^, HAMLET^46^, terpenoids^47^ and antimicrobial peptides^48^. However, our data do not allow us to conclude whether the reported permeabilizing activity of PAβN at concentrations above 20 μg/mL^37^, could contribute to the anti-biofilm activity shown here. Furthermore, we cannot rule out that the reduction in viability observed in our assays could be due, not to bactericidal activity, but to a hypothetical ability of the triple combination to remove biofilm from the coupon surface.

Notably, under our experimental conditions, a treatment frequently used to kill Gram-negative bacterial biofilms colonizing indwelling devices such as catheters (ceftazidime 5000 μg/mL) had an almost negligible anti-biofilm effect (Fig. 4)^49^. This suggests that the experimental combination developed in this work could find application in antibiotic lock therapy (ALT), a strategy aimed at eradicating biofilms formed in the lumen of central venous catheters to salvage these devices and avoid their removal^49^.

The significant toxicity of currently available EPIs has prevented the clinical use of these drugs^15^. In this respect, our results have important implications because we demonstrated that EPI activity can be dramatically enhanced by a permeabilizing molecule and that this potentiation reduces the amount of inhibitor needed to sensitize *P. aeruginosa* to values as low as 1 μg/mL (Tables 2 and 3). However, experimentation in animal models is required to study whether our strategy has therapeutic potential.

Nevertheless, toxicity of current EPIs should not hinder their use in ALT, since the drugs used in this procedure never get into the bloodstream. Interestingly, in the present work, biofilms were exposed to the triple combination for 72 h, whereas ALT solutions are applied for 10–14 days^49^. Furthermore, antibiotic concentrations in ALT solutions are normally adjusted to 1,000 times their planktonic MICs^49^, whereas in our experiments we used a much lower concentration of each agent (i.e. 20 times lower than that value). Thus, the anti-biofilm activity displayed by our experimental therapy is expected to be of much higher magnitude when used for ALT.

## Material and Methods

### Culture conditions and susceptibility testing

The *P. aeruginosa* strains used in this study were the wild type PAO1, the clinical strain Ps4, the MexAB-OprM overexpressing mutant LC1-6 and the PAO1 derivate with a deletion in the efflux pump MexAB-OprM that abrogates its activity K1119^24,26,27^. For routine procedures, bacteria were grown at 37 °C in Tryptic Soy Broth (TSB; BioMerieux) or in TSB supplemented with 16 g/L agar (TSA; Pronadisa, Spain). TSB was also used as medium for biofilm growth. For planktonic cultures, MICs of antimicrobials were determined in Mueller-Hinton cation adjusted (MHCA) broth (Difco Laboratories) using serial twofold dilutions according to CLSI guidelines^50^, as detailed previously^24^. Antimicrobials with MICs higher than the maximum concentration tested were assigned a MIC twice that concentration. MHCA broth was also used for synergy testing (checkerboard assay), growth inhibition experiments (see below) and to test the bactericidal effect of the antimicrobials on biofilm cells.

### Antimicrobial agents

PMBN, Amoxicillin, Ampicillin, Aztreonam, Ceftazidime, Ciprofloxacin, Doxycycline, Erithromycin, Levofloxacin, Ofloxacin, Piperacillin, Tetracycline, Ticarcillin, PAβN and NMP were purchased from Sigma-Aldrich. Azythromycin was obtained from Pfizer (Zithromax® intravenous solution; Viena, Austria). Stock solutions at 10 mg/mL were dissolved as detailed in Supplementary Table 5, according to manufacturer’s recommendations. Then, they were diluted in water and then during the synergy testing, they were further diluted in MHCA.

### Real Time quantitative PCR (RT q-PCR)

The expression of the genes *mexB* and *ampC* was quantified by RT q-PCR according to previously described protocols^51^. Briefly, mid-log phase bacterial cultures in Luria-Bertani broth were collected and total RNA was extracted with the RNeasy Mini kit (Qiagen), treated with DNase (DNA-free DNase Treatment; Ambion), and 0.8 μg were retrotranscripted with the SuperScript kit III (Invitrogen). The cDNA of the gene *mexB* was relatively quantified by RT-qPCR with the SYBR Green PCR Master Mix (Applied Biosystems) following manufacturer’s recommendations and referred to the housekeeping gene *proC* and the reference strain PAO1. The primers used for *mexB* amplification were forward 5′-TTGATAAGGCCCATTTTCGCGT-3′ and reverse 5′-TCTGCTGCTCGATCACCTGGA-3′ with a product size of 310 bp, for *proC* amplification, forward 5′-CAGGCCGGGCAGTTGCTGTC-3′ and reverse 5′-GGTCAGGCGCGAGGCTGTCT-3′ with a product size of 188 bp and for ampC amplification forward 5′-GGCGACATGACAGGGCCT-3′ and reverse 3′-TCCAGGCCGCTGAGGATGGC-5′ with a product size of 296 bp. A relative expression of the genes *mexB* and *ampC* greater than or equal to 3 or 10 respectively, was considered overexpression, as described by Cabot *et al*.^51^. The experiment was repeated independently at least three times.

### Two and three-dimensional synergy testing

Potential synergistic interactions between two antimicrobials were assessed by the checkerboard assay using MHCA broth as described before^25^. To quantify synergistic interactions between three antimicrobials a three dimensional checkerboard test was used^52^. For the latter method, the checkerboard assay was performed in the presence of a fixed concentration of one of the antimicrobials. Briefly, a fresh culture of *P. aeruginosa* Ps4, K1119, PAO1 or LC1-6 was adjusted to 0.5 McFarland standard (equivalent to 10^8^ CFU/mL) and diluted 1:100 with MHCA to obtain a 10^6^ CFU/mL suspension. Aliquots of 100 μL of this suspension were transferred into the wells of a standard microtiter plate and mixed with an equal volume of antimicrobial solution. For each strain, the antibiotic concentration range was selected according to previously determined MICs. In total, 10 different concentrations of the selected antibiotic (Aztreonam, Azithromycin, Ceftazidime, Doxycycline, Levofloxacin or Piperacillin) were combined with 5 different concentrations of the EPI (NMP or PAβN) maintaining PMBN concentration. The concentration of PMBN was adjusted to 1 μg/mL, since preliminary experiments showed that this was the minimum amount of enhancer needed to potentiate the majority of antibiotics in the absence of EPI. Microplates were incubated at 37 °C and growth in the wells was visually assessed after 18–20 h. Each assay included growth control wells containing inoculated medium without antimicrobials and sterility control wells consisting of uninoculated medium. The fractional inhibitory concentration index (FICI) for each double (Equation (1)) or triple (Equation (2)) antimicrobial combination was calculated as follows^53^.

1 $$FIC{I}_{A/B}=\frac{MI{C}_{A(combination)}}{MI{C}_{A(alone)}}+\frac{MI{C}_{B(combination)}}{MI{C}_{B(alone)}}$$

2 $$FIC{I}_{A/B/C}=\frac{MI{C}_{A(combination)}}{MI{C}_{A(alone)}}+\frac{MI{C}_{B(combination)}}{MI{C}_{B(alone)}}+\frac{MI{C}_{C(combination)}}{MI{C}_{C(alone)}}$$

FICIs were calculated with the concentrations in the first non-turbid well found in each row and column of the microplate. Combinations were classified as synergistic (FICI ≤ 0.5), indifferent (0.5 < FICI ≤ 4), and antagonistic (FICI > 4).

### Growth inhibition curves

Kinetics of inhibition of planktonic bacteria by selected antimicrobial combinations was measured in the automated optical analyzer Bioscreen C (Labsystems Laboratories, Helsinki, Finland) in MHCA broth. Bioscreen C monitors the turbidity of bacterial cultures growing in 100-well honeycomb plates at regular intervals. A cell suspension from an overnight culture of Ps4 was first adjusted to OD_600nm_ = 0.040 and then diluted 100 times in the same broth and mixed with the different treatments under study (two and three-component combinations, as well as each antimicrobial alone). Microplate wells were filled with 200 μL of the test suspensions and incubation was carried out at 37 °C for 48 hours with continuous shaking and monitoring the absorbance every 15 minutes at 420–580 nm. Each experiment was independently repeated three times and each concentration was tested in three wells. The inhibitory activity of different treatments was compared by determining the corresponding area under the curve (AUC) during the first 45 h of incubation and by applying the Mann Whitney U Test complemented with Kruskal Wallis comparisons (*p < 0.05).

### Biofilm formation and assessment of anti-biofilm activity

The ability to form biofilm under static conditions in microtiter plates was assessed as described previously^54^.

Biofilms of Ps4 were also grown under dynamic shear conditions using the CDC-reactor (model CBR 90–1, BioSurface Technologies Corporation, Bozeman, MT. USA) as described elsewhere^55^. Briefly, a dense biofilm (1 × 10^12^ CFU/cm^2^, approximately) was developed on the surface of small disks called coupons that were constantly bathed in fresh TSB medium. After 24 h of incubation under continuous flow, coupons were removed from the chamber and planktonic cells were eliminated by rinsing them with phosphate buffer (625 μM KH_2_PO_4_, 2 mM MgCl_2_∙6H_2_O, pH 7.2). Then, the coupons with 48 h mature biofilms attached to their surface were immersed in and treated with 1.75 mL of phosphate buffer, containing either a positive control of bactericidal activity (chlorine 1,000 μg/mL), a standard treatment for Gram-negative biofilms (ceftazidime 5,000 μg/mL)^49^ or different combinations of the antimicrobials at 50 times their planktonic MIC. Finally, coupons were incubated at 37 °C for 72 h with renewal of the solution every 24 h and then they were rinsed with phosphate buffer and processed for colony counting.

For the colony counting method, biofilms were detached by scraping the coupon surface with a sterile wooden stick. Then, biofilm cells were suspended in phosphate buffer, samples were homogenized by sonication for 5 min (Fungilab US1′6; Spain) and aliquots were plated for counting. These count values were used to calculate the so called, log density of the coupon which corresponds to the CFU/cm^2^ of biofilm cells attached to the coupon. In turn, log density allowed the determination of log_10_ reduction, which was defined as the difference of log density between the untreated and the treated biofilm. Experiments were independently repeated twice in duplicate coupons and differences between treated and not treated coupons were analyzed by one-way ANOVA followed by Tukey’s multiple comparison test (*p < 0.05).

For the microscopic assessment of biofilm formation, biofilms grown on coupons were first stained with the LIVE/DEAD BacLight kit (Life Technologies) following the manufacturer’s recommendations. Then, the coupon surface was examined with a Nikon Eclipse E800 fluorescence microscope using the FITC and TRITC filters and the 10x or 20x objectives. Alternatively, microscopic images were taken by confocal laser microscopy (Leica TCS-SP5) using a 1.5x objective and Imaris® software (Bitplane, Switzerland)^55^. Finally, 5 μm thick cryosections (Leica CM1850 cryostat) were obtained as previously described and examined by fluorescence microscopy as mentioned above^56^.

## Supplementary information

Supplementary material
