# Supplementary material for: A permeability-increasing drug synergizes with bacterial efflux pump inhibitors and restores susceptibility to antibiotics in multi-drug resistant Pseudomonas aeruginosa strains
Source: Sci Rep. 2019 Mar 5;9:3452. doi: 10.1038/s41598-019-39659-4 (PMC6401119; doi:10.1038/s41598-019-39659-4)
Supplement: Supplementary file 1 — Supplementary material [file 41598_2019_39659_MOESM1_ESM.doc]

**A permeability-increasing drug synergizes with bacterial efflux pump inhibitors and restores susceptibility to antibiotics in multi-drug resistant *Pseudomonas aeruginosa* strains**

Raquel Ferrer-Espada^a^, *^, 1^, Hawraa Shahrour^a, b, c^, Betsey Pitts^d^, Philip S. Stewart^d^, Susana Sánchez-Gómez^e^, Guillermo Martínez-de-Tejada^a^

^a^ University of Navarra, Department of Microbiology and Parasitology, Irunlarrea 1, 31008 Pamplona, Spain. Navarra Institute for Health Research (IdiSNA)

^b^ Laboratory of Microbiology, Department of Life & Earth Sciences, Faculty of Sciences I, Lebanese University, Hadat campus, Beirut, Lebanon

^c^ Platform of Research and Analysis in Environmental Sciences (PRASE), Doctoral School of Sciences and Technologies, Lebanese University, Hadat campus, Beirut, Lebanon.

^d^ Center for Biofilm Engineering, Montana State University, Bozeman, MT, USA.

^e^ Bionanoplus S.L. Polígono Mocholí. Plaza Cein Nº5, nave B14. 31110 Noain, Navarra, Spain.

* Corresponding author. University of Navarra, Department of Microbiology and Parasitology, Irunlarrea 1, 31008 Pamplona, Spain. Navarra Institute for Health Research (IdiSNA). rfespada2(at)gmail.com +1(857)285-0837

^1^ Present address: Wellman Center for Photomedicine, Massachusetts General Hospital, Harvard Medical School, 55 Fruit Street, Boston, MA, USA 02114.

**Supplementary Table 1.- PMBN sensitizes a MexAB-OprM overexpressing *P. aeruginosa* strain (LC1-6), to EPIs (NMP or PAβN) and antibiotic substrates of MexAB-OprM, as determined by the checkerboard method**.

(a) PAβN

| PAβN^1^ (μg/mL) |  | MIC of Azithromycin (μg/mL) in the presence of the indicated amount of PMBN^2^ | | |  | FICI^3^ |  | MIC of Doxycycline (μg/mL) in the presence of the indicated amount of PMBN | | |  | FICI |  | MIC of Levofloxacin (μg/mL) in the presence of the indicated amount of PMBN | | |  | FICI |  | MIC of Ceftazidime (μg/mL) in the presence of the indicated amount of PMBN | | |  | FICI |  | MIC of Piperacilin (μg/mL) in the presence of the indicated amount of PMBN | | |  | FICI |  | MIC of Aztreonam (μg/mL) in the presence of the indicated amount of PMBN | | |  | FICI |
| --- | --- | --- | --- | --- | --- | --- | --- | --- | --- | --- | --- | --- | --- | --- | --- | --- | --- | --- | --- | --- | --- | --- | --- | --- | --- | --- | --- | --- | --- | --- | --- | --- | --- | --- | --- | --- |
|  |  | 0 |  | 1 μg/mL |  |  |  | 0 |  | 1 μg/mL |  |  |  | 0 |  | 1 μg/mL |  |  |  | 0 |  | 1 μg/mL |  |  |  | 0 |  | 1 μg/mL |  |  |  | 0 |  | 1 μg/mL |  |  |
| 0 |  | 128 |  | 1 |  |  |  | 64 |  | 1 |  |  |  | 2 |  | 0.25 |  |  |  | 4 |  | 4 |  |  |  | 16 |  | 16 |  |  |  | 32 |  | 16 |  |  |
| 1 |  | 256 |  | 0.06 |  | **0.002** |  | 64 |  | 0.12 |  | **0.004** |  | 2 |  | 0.06 |  | **0.032** |  | 4 |  | 0.25 |  | **0.064** |  | 16 |  | 4 |  | **0.252** |  | nd^4^ |  | nd |  | nd |
| 2 |  | 256 |  | 0.003 |  | **0.003** |  | 64 |  | 0.06 |  | **0.004** |  | 2 |  | 0.007 |  | **0.006** |  | 4 |  | 0.015 |  | **0.007** |  | 16 |  | 0.06 |  | **0.007** |  | 16 |  | 16 |  | **0.50** |
| 4 |  | 256 |  | 0.007 |  | **0.005** |  | 64 |  | 0.06 |  | **0.006** |  | 2 |  | 0.015 |  | **0.012** |  | 4 |  | 0.015 |  | **0.009** |  | 16 |  | 0.50 |  | **0.036** |  | 16 |  | 2 |  | **0.07** |
| 8 |  | 256 |  | 0.25 |  | **0.011** |  | 64 |  | 0.12 |  | **0.011** |  | 2 |  | 0.03 |  | **0.024** |  | 4 |  | 0.12 |  | **0.039** |  | 16 |  | 4 |  | **0.259** |  | 16 |  | 4 |  | **0.13** |
| 16 |  | 128 |  | 1 |  | **0.024** |  | 8 |  | 0.50 |  | **0.024** |  | 1 |  | 0.06 |  | **0.047** |  | 1 |  | 0.50 |  | **0.142** |  | 16 |  | 8 |  | 0.517 |  | 16 |  | 4 |  | **0.14** |

(b) NMP

| NMP^5^ (μg/mL) |  | MIC of Azithromycin (μg/mL) in the presence of the indicated amount of PMBN | | |  | FICI |  | MIC of Doxycycline (μg/mL) in the presence of the indicated amount of PMBN | | |  | FICI |  | MIC of Levofloxacin (μg/mL) in the presence of the indicated amount of PMBN | | |  | FICI |  | MIC of Ceftazidime (μg/mL) in the presence of the indicated amount of PMBN | | |  | FICI |  | MIC of Piperacilin (μg/mL) in the presence of the indicated amount of PMBN | | |  | FICI |  | MIC of Aztreonam (μg/mL) in the presence of the indicated amount of PMBN | | |  | FICI |
| --- | --- | --- | --- | --- | --- | --- | --- | --- | --- | --- | --- | --- | --- | --- | --- | --- | --- | --- | --- | --- | --- | --- | --- | --- | --- | --- | --- | --- | --- | --- | --- | --- | --- | --- | --- | --- |
|  |  | 0 |  | 1 μg/mL |  |  |  | 0 |  | 1 μg/mL |  |  |  | 0 |  | 1 μg/mL |  |  |  | 0 |  | 1 μg/mL |  |  |  | 0 |  | 1 μg/mL |  |  |  | 0 |  | 1 μg/mL |  |  |
| 0 |  | 128 |  | 1 |  |  |  | 64 |  | 1 |  |  |  | 2 |  | 0.25 |  |  |  | 4 |  | 4 |  |  |  | 16 |  | 16 |  |  |  | 32 |  | 16 |  |  |
| 1 |  | >256 |  | 0.50 |  | **0.02** |  | 64 |  | 1 |  | **0.03** |  | 2 |  | 0.25 |  | **0.14** |  | 8 |  | 2 |  | 0.52 |  | 16 |  | 16 |  | 1.02 |  | nd |  | nd |  | nd |
| 2 |  | >256 |  | 0.25 |  | **0.03** |  | >64 |  | 1 |  | **0.05** |  | 2 |  | 0.25 |  | **0.16** |  | 4 |  | 2 |  | 0.53 |  | 16 |  | 16 |  | 1.03 |  | 16 |  | 16 |  | 0.53 |
| 4 |  | >256 |  | 0.25 |  | **0.07** |  | 64 |  | 0.50 |  | **0.07** |  | 2 |  | 0.12 |  | **0.12** |  | 4 |  | 2 |  | 0.56 |  | 16 |  | 16 |  | 1.06 |  | 16 |  | 16 |  | 0.56 |
| 8 |  | >256 |  | 0.50 |  | **0.13** |  | 32 |  | 0.50 |  | **0.13** |  | 2 |  | 0.12 |  | **0.19** |  | 4 |  | 1 |  | **0.38** |  | 32 |  | 8 |  | 0.63 |  | 16 |  | 16 |  | 0.63 |
| 16 |  | >256 |  | 1 |  | **0.26** |  | 64 |  | 0.50 |  | **0.26** |  | 2 |  | 0.12 |  | **0.31** |  | 4 |  | 0.50 |  | **0.38** |  | 16 |  | 4 |  | **0.50** |  | 16 |  | 8 |  | **0.50** |

^1^: MIC _PAβN_ >512 μg/mL. ^2^: MIC _PMBN_ >512 μg/mL. ^3^: Fractional Inhibitory Concentration Index. ^4^: not determined. ^5^: MIC _NMP_ =64 μg/mL. Synergistic combinations are indicated in bold.

**Supplementary Table 2.- PMBN sensitizes a MexAB-OprM overexpressing *P. aeruginosa* clinical strain (Ps4) to EPIs (NMP or PAβN) and antibiotic substrates of MexAB-OprM, as determined by the checkerboard method**.

(a) PAβN

| PAβN^1^ (μg/mL) |  | MIC of Azithromycin (μg/mL) in the presence of the indicated amount of PMBN^2^ | | |  | FICI^3^ |  | MIC of Doxycycline (μg/mL) in the presence of the indicated amount of PMBN | | |  | FICI |  | MIC of Ceftazidime (μg/mL) in the presence of the indicated amount of PMBN | | |  | FICI |  | MIC of Piperacillin (μg/mL) in the presence of the indicated amount of PMBN | | |  | FICI |
| --- | --- | --- | --- | --- | --- | --- | --- | --- | --- | --- | --- | --- | --- | --- | --- | --- | --- | --- | --- | --- | --- | --- | --- | --- |
|  |  | 0 |  | 1 μg/mL |  |  |  | 0 |  | 1 μg/mL |  |  |  | 0 |  | 1 μg/mL |  |  |  | 0 |  | 1 μg/mL |  |  |
| 0 |  | 128 |  | 64 |  |  |  | 64 |  | 32 |  |  |  | 64 |  | 32 |  |  |  | 256 |  | 128 |  |  |
| 1 |  | 256 |  | 64 |  | **0.50** |  | 64 |  | 16 |  | **0.25** |  | 64 |  | 32 |  | **0.50** |  | 256 |  | 128 |  | **0.50** |
| 2 |  | 256 |  | 32 |  | **0.25** |  | 64 |  | 8 |  | **0.13** |  | 64 |  | 32 |  | **0.50** |  | 256 |  | 128 |  | **0.50** |
| 4 |  | 128 |  | 0.25 |  | **0.01** |  | 64 |  | 0.50 |  | **0.01** |  | 64 |  | 8 |  | **0.13** |  | 256 |  | 32 |  | **0.13** |
| 8 |  | 256 |  | 0.25 |  | **0.01** |  | 32 |  | 1 |  | **0.02** |  | 64 |  | 4 |  | **0.07** |  | 256 |  | 64 |  | **0.26** |
| 16 |  | 32 |  | 1 |  | **0.02** |  | 4 |  | 1 |  | **0.03** |  | 64 |  | 16 |  | **0.27** |  | 256 |  | 128 |  | 0.52 |

(b) NMP

| NMP^4^  (µg/mL) |  | MIC of Azithromycin (μg/mL) in the presence of the indicated amount of PMBN^2^ | | |  | FICI |  | MIC of Doxycycline (μg/mL) in the presence of the indicated amount of PMBN | | |  | FICI |  | MIC of Ceftazidime (μg/mL) in the presence of the indicated amount of PMBN | | |  | FICI |  | MIC of Piperacillin (μg/mL) in the presence of the indicated amount of PMBN | | |  | FICI |
| --- | --- | --- | --- | --- | --- | --- | --- | --- | --- | --- | --- | --- | --- | --- | --- | --- | --- | --- | --- | --- | --- | --- | --- | --- |
|  |  | 0 |  | 1 μg/mL |  |  |  | 0 |  | 1 μg/mL |  |  |  | 0 |  | 1 μg/mL |  |  |  | 0 |  | 1 μg/mL |  |  |
| 0 |  | 256 |  | 64 |  |  |  | 64 |  | 32 |  |  |  | 64 |  | 32 |  |  |  | 256 |  | 128 |  |  |
| 1 |  | 256 |  | 16 |  | **0.07** |  | 64 |  | 0.25 |  | **0.01** |  | 32 |  | 16 |  | **0.26** |  | 128 |  | 8 |  | **0.04** |
| 2 |  | 256 |  | 4 |  | **0.03** |  | 64 |  | 0.25 |  | **0.02** |  | 32 |  | 4 |  | **0.08** |  | 128 |  | 8 |  | **0.05** |
| 4 |  | 256 |  | 4 |  | **0.05** |  | 64 |  | 0.125 |  | **0.03** |  | 32 |  | 0.25 |  | **0.04** |  | 128 |  | 4 |  | **0.05** |
| 8 |  | 256 |  | 0.25 |  | **0.06** |  | 64 |  | 0.063 |  | **0.06** |  | 32 |  | 0.063 |  | **0.06** |  | 128 |  | 0.25 |  | **0.06** |
| 16 |  | 256 |  | 0.125 |  | **0.13** |  | 64 |  | 0.063 |  | **0.13** |  | 16 |  | 0.063 |  | **0.13** |  | 32 |  | 0.25 |  | **0.13** |

^1^: MIC _PAβN_ >512 μg/mL. ^2^: MIC _PMBN_>512 μg/mL. ^3^: Fractional Inhibitory Concentration Index. ^4^: MIC _NMP_ = 128 μg/mL Synergistic combinations are indicated in bold.

**Supplementary Table 3.- Synergy testing conducted on *Pseudomonas aeruginosa* K1119 by the checkerboard method.**

(a) PAβN

| PAβN^1^ (µg/mL) |  | MIC of Azithromycin (μg/mL) in the presence of the indicated amount of PMBN^2^ | | |  | FICI^3^ |  | MIC of Doxycycline (μg/mL) in the presence of the indicated amount of PMBN | | |  | FICI |  | MIC of Piperacillin (μg/mL) in the presence of the indicated amount of PMBN | | |  | FICI |
| --- | --- | --- | --- | --- | --- | --- | --- | --- | --- | --- | --- | --- | --- | --- | --- | --- | --- | --- |
|  |  | 0 |  | 1 µg/mL |  |  |  | 0 |  | 1 µg/mL |  |  |  | 0 |  | 1 µg/mL |  |  |
| 0 |  | 32 |  | 16 |  |  |  | 2 |  | 0.125 |  |  |  | 0.5 |  | 0.5 |  |  |
| 1 |  | 16 |  | 16 |  | 0.502 |  | 2 |  | 0.125 |  | **0.064** |  | 0.5 |  | 0.5 |  | 1.002 |
| 2 |  | 16 |  | 16 |  | 0.503 |  | 2 |  | 0.125 |  | **0.065** |  | 0.5 |  | 0.5 |  | 1.003 |
| 4 |  | 16 |  | 16 |  | 0.505 |  | 2 |  | 0.125 |  | **0.067** |  | 0.5 |  | 0.5 |  | 1.005 |
| 8 |  | 16 |  | 16 |  | 0.509 |  | 2 |  | 0.125 |  | **0.071** |  | 0.5 |  | 0.5 |  | 1.009 |
| 16 |  | 16 |  | 16 |  | 0.517 |  | 2 |  | 0.125 |  | **0.079** |  | 0.5 |  | 0.5 |  | 1.017 |

(b) NMP

| NMP^4^ (µg/mL) |  | MIC of Azithromycin (μg/mL) in the presence of the indicated amount of PMBN | | |  | FICI |  | MIC of Doxycycline (μg/mL) in the presence of the indicated amount of PMBN | | |  | FICI |  | MIC of Piperacillin (μg/mL) in the presence of the indicated amount of PMBN | | |  | FICI |
| --- | --- | --- | --- | --- | --- | --- | --- | --- | --- | --- | --- | --- | --- | --- | --- | --- | --- | --- |
|  |  | 0 |  | 1 µg/mL |  |  |  | 0 |  | 1 µg/mL |  |  |  | 0 |  | 1 µg/mL |  |  |
| 0 |  | 32 |  | 16 |  |  |  | 2 |  | 0.125 |  |  |  | 0.5 |  | 0.5 |  |  |
| 1 |  | 32 |  | 16 |  | 0.517 |  | 2 |  | 0.125 |  | **0.079** |  | 0.5 |  | 0.5 |  | 1.017 |
| 2 |  | 32 |  | 8 |  | **0.282** |  | 1 |  | 0.06 |  | **0.063** |  | 0.5 |  | 0.5 |  | 1.032 |
| 4 |  | 16 |  | 0.03 |  | **0.064** |  | 1 |  | 0.03 |  | **0.079** |  | 0.25 |  | 0.125 |  | **0.313** |
| 8 |  | 0.06 |  | 0.03 |  | **0.127** |  | 0.03 |  | 0.016 |  | **0.134** |  | 0.03 |  | 0.03 |  | **0.188** |
| 16 |  | 0.03 |  | 0.03 |  | **0.252** |  | 0.03 |  | 0.016 |  | **0.259** |  | 0.03 |  | 0.03 |  | **0.313** |

^1^: MIC _PAβN_ >512 μg/mL. ^2^: MIC _PMBN_ >512 μg/mL. ^3^: Fractional Inhibitory Concentration Index. ^4^: MIC _NMP_ = 64 μg/mL. Synergistic combinations are indicated in bold.

**Supplementary Table 4.- Synergy testing conducted on *Pseudomonas aeruginosa* PAO1 by the checkerboard method.**

(a) PAβN

| PAβN^1^ (µg/mL) |  | MIC of Azithromycin (μg/mL) in the presence of the indicated amount of PMBN^2^ | | |  | FICI^3^ |  | MIC of Doxycycline (μg/mL) in the presence of the indicated amount of PMBN | | |  | FICI |  | MIC of Piperacillin (μg/mL) in the presence of the indicated amount of PMBN | | |  | FICI |
| --- | --- | --- | --- | --- | --- | --- | --- | --- | --- | --- | --- | --- | --- | --- | --- | --- | --- | --- |
|  |  | 0 |  | 1 µg/mL |  |  |  | 0 |  | 1 µg/mL |  |  |  | 0 |  | 1 µg/mL |  |  |
| 0 |  | 256 |  | 4 |  |  |  | 4 |  | 0.03 |  |  |  | 4 |  | 1 |  |  |
| 1 |  | 256 |  | 4 |  | **0.018** |  | 4 |  | 0.03 |  | **0.010** |  | 2 |  | 0.5 |  | **0.127** |
| 2 |  | 256 |  | 4 |  | **0.019** |  | 4 |  | 0.03 |  | **0.011** |  | 2 |  | 0.5 |  | **0.128** |
| 4 |  | 256 |  | 4 |  | **0.021** |  | 2 |  | 0.03 |  | **0.013** |  | 0.5 |  | 0.5 |  | **0.130** |
| 8 |  | 64 |  | 4 |  | **0.024** |  | 0.25 |  | 0.03 |  | **0.017** |  | 0.5 |  | 0.5 |  | **0.134** |
| 16 |  | 32 |  | 4 |  | **0.032** |  | 0.25 |  | 0.03 |  | **0.024** |  | 0.5 |  | 0.5 |  | **0.142** |

(b) NMP

| NMP^4^ (µg/mL) |  | MIC of Azithromycin (μg/mL) in the presence of the indicated amount of PMBN | | |  | FICI |  | MIC of Doxycycline (μg/mL) in the presence of the indicated amount of PMBN | | |  | FICI |  | MIC of Piperacillin (μg/mL) in the presence of the indicated amount of PMBN | | |  | FICI |
| --- | --- | --- | --- | --- | --- | --- | --- | --- | --- | --- | --- | --- | --- | --- | --- | --- | --- | --- |
|  |  | 0 |  | 1 µg/mL |  |  |  | 0 |  | 1 µg/mL |  |  |  | 0 |  | 1 µg/mL |  |  |
| 0 |  | 256 |  | 4 |  |  |  | 4 |  | 0.03 |  |  |  | 4 |  | 1 |  |  |
| 1 |  | 256 |  | 4 |  | **0.032** |  | 4 |  | 0.03 |  | **0.024** |  | 4 |  | 1 |  | **0.267** |
| 2 |  | 256 |  | 0.25 |  | **0.033** |  | 4 |  | 0.03 |  | **0.040** |  | 4 |  | 0.06 |  | **0.048** |
| 4 |  | 256 |  | 0.06 |  | **0.064** |  | 4 |  | 0.008 |  | **0.065** |  | 4 |  | 0.03 |  | **0.071** |
| 8 |  | 0.5 |  | 0.03 |  | **0.126** |  | 0.5 |  | 0.008 |  | **0.128** |  | 0.03 |  | 0.016 |  | **0.130** |
| 16 |  | 0.25 |  | 0.03 |  | **0.251** |  | 0.03 |  | 0.008 |  | **0.253** |  | 0.03 |  | 0.008 |  | **0.253** |

^1^: MIC _PAβN_ >512 μg/mL. ^2^: MIC _PMBN_ >512 μg/mL. ^3^: Fractional Inhibitory Concentration Index. ^4^: MIC _NMP_ = 64 μg/mL. Synergistic combinations are indicated in bold.

**Supplementary Table 5.- Solvents used for the compounds under study.**

| Antimicrobial agent |  | Solvent |
| --- | --- | --- |
| Amoxicillin |  | phosphate buffer pH 6.0, 0.1M |
| Ampicillin sodium salt |  | water |
| Azithromycin |  | stock solution at 100 mg/mL 95% ethanol |
| Aztreonam |  | saturated solution of sodium bicarbonate |
| Ceftazidime hydrate |  | water |
| Ciprofloxacin |  | water |
| Doxycycline hyclate |  | water |
| Erithromycin |  | 50% ethanol, 50% water |
| Levofloxacin |  | water+0.1 M NaOH dropwise to dissolve |
| NMP |  | 10% glacial acetic acid+ 90% water |
| Ofloxacin |  | water +0.1mol/L NaOH dropwise to dissolve |
| PAβN |  | water |
| Piperacillin sodium salt |  | water |
| PMBN |  | water |
| Tetracycline |  | water |
| Ticarcillin disodium salt |  | water |
